# Silkworm (*Bombyx mori* L.) pupae meal supplementation: Effect on growth performance, carcass traits, meat quality, and economic efficiency in rabbits

**DOI:** 10.1007/s11250-026-04863-8

**Published:** 2026-02-02

**Authors:** Hanan A. M. Hassanien, Ayman Hassan, Mohammed E. Gad, Noura Gouda, Hamza M. Kamel, Nabila M. El-Kassas, Mohamed A. Radwan, Usama Nayel, Abdelfattah Z. M. Salem

**Affiliations:** 1 Animal Production Research Institute, Agricultural Research Center, Giza, Egypt; 2 Department of Zoology and Entomology, Faculty of Science, Al-Azhar University, Nasr City, 11884 Cairo Egypt; 3 Department of Animal Production, Faculty of Technology and Development, Zagazig University, Zagazig, Egypt; 4 Plant Protection Department, Faculty of Agriculture, Al-Azhar University, Nasr City, Cairo Egypt; 5 Department of Animal Production, Faculty of Agriculture, Cairo University, Giza, Egypt; 6 Department of Animal Production, Faculty of Agriculture, Menoufia University, Shebin, Elkom 32514 Egypt; 7 Dipartimento di Scienze del Suolo, della Pianta e degli Alimenti (Di.S.S.P.A.), Università degli Studi di Bari, Via Giovanni Amendola, 165/a, Bari, 70126 BA Italy

**Keywords:** *Bombyx mori*, Insect protein, Rabbit nutrition, Silkworm pupae

## Abstract

This study investigated how consuming silkworm pupae (SP) meal affected rabbits that were still growing. Forty-five weaned male New Zealand White rabbits, averaging 680 g, were allocated randomly to three groups: a control group (SP0) and two groups that had SP 0.5% (SP0.5) and 1% (SP1). The SP exhibited a significant enhancement in body weight, average daily increase, and feed conversion ratio (*P* < 0.05). The amount of feed each group consumed stayed the same. The digestibility of dry matter, organic matter, crude fiber, and nitrogen-free extract did not change, but the digestibility of crude protein and ether extract, and the amount of nitrogen retained were all much greater (*P* < 0.05) in rabbits fed SP. There were no significant differences between carcass weight, slaughter body weight, and dressing %. The amount of protein, moisture, and ash in the meat was the same, but the amount of fat was greater (*P* < 0.05) in the SP groups. The pH of the cecum, the levels of ammonia nitrogen, acetate, butyrate, and the acetate-to-propionate ratio stayed the same, but the total volatile fatty acids went up a lot (*P* < 0.05) in rabbits that ate SP. The SP also caused levels of total plasma protein, albumin, and globulin to rise by a lot. The kidneys (urea and creatinine) worked just fine. It was concluded that adding up to 1% dried silkworm pupae to rabbit meals does not hurt their growth or blood parameters.

## Introduction

Researchers in animal production are concentrating on identifying sustainable, nutritious, and cost-effective sources of protein and energy. Silkworm pupae, a by-product of silk production, are predominantly utilized as fertilizer and animal fodder in Southeast Asia (Sheikh et al. 2018; Dewi Apri and Komalasari 2020). The recycling and utilization of these pupae to generate valuable products offer ecological and economic benefits (Herman et al. 2022). Rumpold and Schlüter (2013) emphasised that silkworms are rich in amino acids, energy, protein, fatty acids, biotin, pantothenic acid, and riboflavin, noting that the latter three are essential nutrients. Moreover, 100 g of silkworm eggs have about 10 mg of tocopherols, which are more than found in many popular foods. Particularly, food management is an effective control technique for the mineral and vitamin levels of silkworm pupae raised on farms (Wu et al. 2021). Traditional protein-rich feed sources for monogastric animals, such as soybean meal, are distinguished by having a high n-6 fatty acids concentration. In contrast, silkworm pupae demonstrate an elevated degree of n-3 fatty acids, comparable to those found in linseed and camelina (Hăbeanu et al. 2014; Matthäus and Özcan 2017; Ostrikov et al. 2021). Numerous studies (Fagoonee 1983; Valerie et al. 2015) show that adding 5–10% silkworm pupae to animal diets could be replaced 50% of conventional protein sources, like fish meal or soybean meal. The sole organism that has been entirely domesticated is the mulberry silkworm (*Bombyx mori* L., 1758), which has been cultivated by humans and raised in captivity for around 7,500 years (Yang et al. 2014). Among the various stages of its life cycle, the most often eaten food or feed is silkworm pupae, which make up around 60% of the cocoon’s dry weight (Hu et al. 2017). After silk production, these pupae are typically regarded as waste. For every kilogram of raw silk produced, about 5,500 pupae are generated. These pupae, making up roughly 60% of the cocoon’s dry weight, are mainly used as fertilizer or discarded as industrial waste (Sahib et al. 2024). Byproducts of the production of one kilogram of unprocessed silk included approximately 8.014 kg of moist and 2 kg of dried silkworm pupae. It is important to note that pupae are a promising source of minerals, lipids, and proteins (Tassoni et al. 2022). Dry silkworm pupae contain 25% to 30% fat (Longvah et al. 2011; Kouřimská and Adámková 2016). These pupae have a high level of both monounsaturated and polyunsaturated fatty acids, complemented by a comparatively low concentration of saturated fats (Payne et al. 2016). Unsaturated fatty acids in silkworms pupae are between 70% and 80%, with 1% containing unsaponifiable matter, which contains substances like cholesterol, β-sitosterol, and campesterol. Notably, silkworm pupae are mainly abundant in α-linolenic acid, which constitutes approximately 71% of their fatty acid profile (Rao 1994; Pereira et al. 2003; Zhao et al. 2015a, b). As such, they serve as a valuable source of functional fatty acids. Additionally, the oxidative stability of their total lipid content is remarkably elevated, primarily attributable to the synergistic interactions of tocopherols and phospholipids, which proficiently impede lipid oxidation (Kotake-Nara et al. 2002). Silkworm pupae serve as a remarkable source of calcium and iron, showcasing a notable potassium content of 34.0 mg/g, complemented by a low sodium-to-potassium ratio of 0.08. Their zinc content is notably substantial, measuring at 36 µg/g (Zhou and Han 2006; Wu et al. 2021). Furthermore, silkworm proteins and hydrolyzed peptides have demonstrated a range of functions, such as bolstering immunity and displaying antitumor and antioxidant characteristics (Wu et al. 2021). Hydrolysed peptides sourced from silkworm pupae possess a range of physiological advantages. The residues of silkworm pupae consist of 2% to 8% chitosan, around 4% polysaccharides, and minimal quantities of antibacterial peptides along with other bioactive substances, each exhibiting a range of biological activities (Wang et al. 2007; Luo et al. 2010; Yue et al. 2013; Battampara et al. 2020). Silkworm pupae serve as a significant substitute for fishmeal in the nutrition of poultry, fish, and crustaceans (Konwar et al. 2008; Sun et al. 2014; Rahimnejad et al. 2019). Incorporating silkworm pupae into animal feed may help reduce reliance on traditional protein sources while positively affecting moulting times, enhancing antioxidant capacity, and improving digestibility. Additionally, besides human consumption, silkworm pupae act as a valuable source of nutrition in animal feed. Silkworm pupae meal has a high protein content, which is used as a protein source for livestock, notably monogastric species (poultry, pigs, and fish), as well as ruminants (Trivedy et al. 2008). Liu et al. (1987) highlight the applicability of the inclusion of silkworm pupae in animal feed. Carregal and Takahashi (1987), Gugołek et al. (2019), and Kowalska et al. (2020) explored the suitability of silkworm pupae as an alternative to soybean meal in rabbit rations. The main objective of this study is to assess the integration of silkworm pupae in rabbit rations on growth efficiency, blood parameters, carcass features, meat quality, and economic efficiency.

## Materials and methods

### Silkworm pupae

**Fig. 1:**
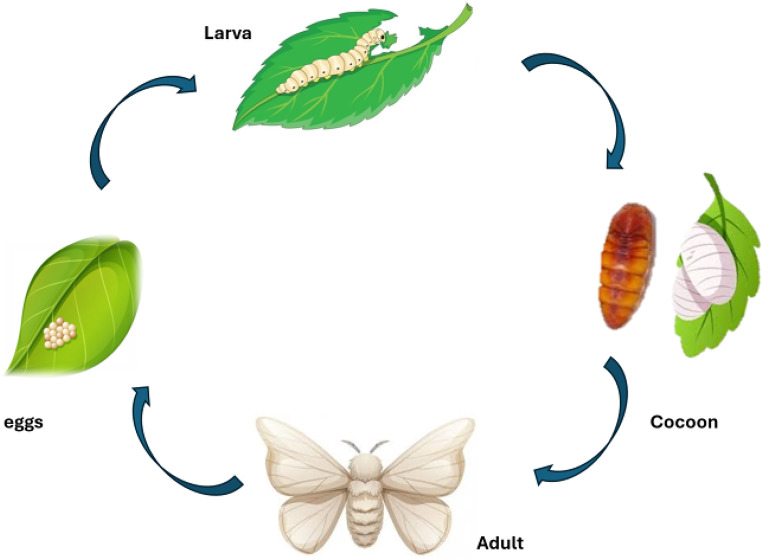
Life cycle of the silkworm

Bombyx mori larvae were obtained from the Department of Plant Protection’s lab at the Faculty of Agriculture, Al-Azhar University, Nasr City, Cairo, Egypt. The rearing chamber and facilities were completely cleaned in preparation for rearing, which involved washing the floor with a 5% bleaching powder solution. Subsequently, the entire room was disinfected using a spray of 2.5% Sanitech in a 0.5% slaked lime solution (Dandin and Jayaswal Jayant 2003). Eggs of the Thio1 hybrid silkworm were imported from Thailand, and the rearing process was carried out under hygienic conditions, at a steady state temperature of 26 ± 2 °C and 70 ± 5% relative humidity, by the guidelines established by Krishnaswami (1986). During the spring season of 2023, the eggs were incubated at 24 °C and 80% relative humidity. To ensure conditions for the young larvae (1st -3rd instars), the newly hatched larvae were covered with plastic sheets and surrounded by moist sponge strips. They were fed Morus alba var. mulberry leaves until they reached the pupal stage. After each molt, cleaning nets with holes measuring 3 × 3 mm were utilized to remove dried leaves and feces during the early larval stages. Following the fifth larval stadium, the larvae ceased feeding and began the process of spinning cocoons. After ten days, the cocoons were gathered using collapsible frames to help the spinning process (Fig. 1).

### Feeding and management

Forty-five male New Zealand white (NZW) rabbits aged 42 days (with an average body weight of about 680.45 ± 42.67 g) were used in this study. The rabbits were randomly allocated to three treatment groups (equal groups) with five replicates for *n* = 3 for each replicate. The rabbit’s diet included a control group (SP0) with neglected baseline diets and experimental groups’ silkworm pupae meal at 0.5% (SP0.5) and 1% (SP1). Throughout the 56-day study, each cage had feeding hoppers and drinking nipples, providing the rabbits with uninterrupted access to food and clean water. The animals were prepared with commercial pelleted feed manufactured to NRC (1977) specifications. The recipe for the diet under study is explained in Table 1. Individual weights were measured weekly to assess the final body weight (FBW), and daily records of feed consumption and leftovers per cage were kept, calculating average daily feed intake. Moreover, the average daily weight gain (ADG) and the feed conversion ratio (FCR g feed/g gain) were calculated. The daily death rates for each group were documented along with the percentage assigned post–experiment.

**Table 1:** Ingredients and chemical composition of the experimental diet fed and silkworm meal

Ingredients	% of DM	Chemical composition, g/100 g DM^2^		
		Item	Diet	Silkworm meal
Berseem hay	30	DM	91.84	88.57
Barley grain	18.8	OM	93.19	89.27
Yellow corn	5.3	CP	16.81	56.92
Dried sugar beet pulp	5	CF	14.54	5.16
Wheat bran	13.5	EE	3.12	24.77
Soybean meal	20.4	NFE	58.72	2.42
Molasses	3.6	Ash	6.81	10.73
Dicalcium phosphate	1.4	NDF	42.75	6.36
Limestone	1.1	ADF	23.62	5.22
Salt	0.5	ADL	4.66	2.41
Premix^1^	0.3	DE, Mcal /kg	2795.6	1896.1
Methionine	0.1			

^1^ Each kg of premix contained 100 mg vitamin E, 10 mg vitamin B1, 20 mg, vitamin B2, 400,000 IU vitamin A, 100,000 IU vitamin D, 30 g Calcium, 12 g Phosphorus, 40 g Na, 1000 mg Cu, 60 mg I, 60 mg Co, 11 g Mg, 2000 mg Manganese, 2000 mg Zn, 3000 mg Fe

^2^DM: Dry matter; OM: organic matter; CP: crude protein; CF: crude fibre; EE: ether extract; NFE: nitrogen-free energy; NDF: neutral detergent fibre; ADF: acid detergent fibre; ADL: acid detergent lignin; DE: digestible energy

### Digestibility trial

The nutritional digestibility coefficients were measured in a digestibility trial, following the approach set by Perez et al. (2010). During a one-week adaptation period, three rabbits from each group were housed individually in metabolic cages and fed the experimental ration. Following this initial stage, faeces samples were collected daily, 24 h before breakfast, for 5 the following days during the entire monitoring period. Additionally, the daily faeces samples were collected from individual rabbits, which were then oven-dried at 70 °C for 48 h, ground into a powder, and kept for further proximate chemical analysis. As per AOAC (2005), both feed and dried faecal samples were evaluated for dry matter (DM), crude protein (CP), crude fiber (CF), nitrogen-free extract (NFE), neutral detergent fiber (NDF), and acid detergent fiber (ADF).

### Carcass traits

From each group, five rabbits were randomly chosen and subjected to a 12-hour starvation period. To evaluate the carcass characteristics, each rabbit was weighed and humanely dispatched via jugular vein severance. To assess dressing percentage (slaughter weight over body weight), measurements were taken post-haemorrhage weighting. Furthermore, additional weight measurements of the liver, kidneys, and heart were taken. In preparation for chemical analysis, the viscera were crushed individually, and samples of feed and excrement were also processed using a Cyclotec mill (Cyclotec 1093, Foss, Germany) with a 1-mm screen. The moisture content was determined by drying the samples in a 70 °C oven until they reached a consistent weight. The CP content was determined by Kjeldahl’s procedure (technique No. 978.04, AOAC 2005). The Soxhlet extraction method was employed to evaluate the ether extract, with petroleum ether serving as the extracting agent at a temperature range of 60–80 °C (Method No. 930.09, AOAC 2005). The samples were incinerated in a muffle furnace at 550 °C to evaluate their ash (Method No. 930.05, AOAC 2005). The Tecator Fibretic System was employed to analyze the contents of NDF, ADF, and acid detergent lignin (ADL) in accordance with the methodology outlined by Van Soest et al. (1991).

### Cecum fermentation

Following the euthanasia of the rabbits, the cecal contents were carefully extracted and placed in a sterile beaker together with 10 ml of distilled water, 5 ml of saturated sulfuric acid (1.5 M H_2_SO_4_), and 5 ml of magnesium sulfate (1–5 M). Then the mixture was incubated at 4 °C for 24 h prior to filtering via two layers of sterile gauze. The final liquid’s pH was measured by a digital pH meter. After centrifuging the contents of cecal at 7,000 × g for 10 min at 20 °C, the supernatant was divided into two equal amounts. The ammonia nitrogen (NH_3_-N) concentration was assayed by treating the first part with a 0.2 M solution of hydrochloric acid, using 1 mL of HCl for every 1 mL sample (v/v). Volatile fatty acid (VFA) were measured by treating the second aliquot with orthophosphoric acid 5% (v/v) and mercuric chloride 1% (w/v) (0.1 mL/mL of the sample). High-performance liquid chromatography (HPLC; Model Water 600; UV detector, Millipore Corp., USA) was conducted to determine the molar concentrations of acetic, propionic, and butyric acids, according to the procedure of Samuel et al. (1997).

### Blood sampling and analysis

Three millilitres of blood were collected in heparinised tubes from the rabbits after they had been anaesthetised and stored in an icebox. Then all samples were centrifuged at 5000 rpm for 10 min, then the supernatant was decanted and kept at -20 °C till analysed. The blood parameters in terms of glucose, cholesterol, triglycerides, high-density lipoprotein (HDL), low-density lipoprotein (LDL), urea, creatinine, Aspartate Transaminase (AST), and Alanine Transaminase (ALT) were measured using colorimetric techniques using standard kits from Bio-Merieux, a French company. In order to evaluate the total antioxidant capacity (TAC), superoxide dismutase (SOD), glutathione peroxidase (GPx), glutathione reductase (GR), and thiobarbituric acid reactive substances, the serum was also kept at -20 °C. Commercial kits from Bio Diagnostic Company (Giza, Egypt) and a Optizen Pop spectrophotometer (Mecasys, Korea) were used.

### Statistical analysis

Data were analysed using the General Linear Model procedure by SAS (2002), which was conducted to analyse data from the totally randomised design. A statistical model is as follows: Y_ij_ = µ + d_i_ + ε_ij_. The measured value is Y_ij_, µ: the overall mean, d_i_ is the i^th^ diet’s impact, and ε_ij_: the random error associated with the j^th^ rabbit on the i^th^ diet in this example. At a significance threshold of *P* < 0.05, Duncan’s multiple range test was operated to evaluate the significance of variations in means (Duncan 1955).

## Results and discussion

In recent decades, researchers across several fields have devoted a major effort to using all available resources and sustainably managing waste. Increasing environmental concerns and resource shortages have prompted power companies are acquire innovative ways to minimize waste and utilize byproducts effectively. Silkworm was used widely in different forms like meal, defatted meal oil, protein, or pupae (Rodríguez-Ortiz et al. 2024). Moreover, it is used in different industries such as human food, human health, animal feed, and veterinary. The purpose of this research was to evaluate the effects of adding SP to rabbit diets on meat quality, carcass features, digestibility, productivity, and health.

### Chemical composition of silkworm pupae and diets

Table 1 presents the chemical composition of the experimental silkworm pupae meal and the diet. Most nutrients exhibited a significant increase in SP. The data indicated that SP’s CP content, EE, and ash content displayed values similar to those documented by Gugolek et al. (2019). Finke (2002) and Usub et al. (2008), reported reduced CP content levels in their studies.

### Productive performance

Table 2 demonstrates that the final body weight, average daily gain, and feed conversion ratio were substantially elevated in the rabbit groups administered SP (SP0.5 and SP1) *versus* the control group (SP0) (*P* < 0.05). No substantial differences were seen in total feed consumption across the groups. These findings may be attributed to its SP concentration for crude protein and ether extracts, as seen in Table 3 where groups fed on SP enhance protein digestibility and nitrogen efficiency, promoting overall nutrient utilization. Where the contents were significantly affected by the inclusion of SP in the feed (Rodríguez-Ortiz et al. 2024). While adding SP to rabbit diets at or below 10% had no effect on energy, neutral detergent fiber, acid detergent fiber, crude protein, dry matter, organic matter, or acid detergent lignin digestibility, Gugołek et al. (2021) found that the small intestine’s digestive viscosity was negligible. The growth performance metrics and carcass features of broiler and laying hens were unaffected by the addition of SP to their diets, as reported by Priyadharshini et al. (2017) and Miah et al. (2020). When intestinal contents are thicker, peristaltic mixing of digesta is less efficient, bile acid production is reduced, and deconjugation activities are hindered. Because of this, nutrients have a harder time passing through the intestinal barrier, which slows down digestion and makes it harder for the body to absorb organic nutrients (Konieczka and Smulikowska 2018).

**Table 2:** Effect of silkworm (*Bombyx mori* L.) pupae meal supplementation on growth performance of growing rabbits

Items	Experimental groups^1^			SEM^2^	*P*-value^3^
	SP0	SP0.5	SP1		
Initial body weight, g	687.46	676.67	677.20	86.35	0.842
Final body weight, g	2051.0^b^	2180.3^a^	2197.0^a^	52.77	0.031
Average daily gain, g	24.34^b^	26.85^a^	27.14^a^	0.28	0.026
Total feed intake, g	109.35	112.04	114.10	14.86	0.873
Feed conversion ratio, g feed/g gain	4.73 ^a^	4.42^b^	4.55^b^	0.14	0.012

^1^ Control: SP0; SP0.5: 0.5% Silkworm meal: SP1; 1.0% Silkworm meal

^2^ SEM: standard error of mean

^3^ In the rows, (*P* < 0.05) different letters (a and b) indicate statistically significant differences between means

**Table 3:** Effect of silkworm (*Bombyx mori* L.) pupae meal supplementation on digestibility coefficients, nutritive values, and nitrogen utilization of growing rabbits

Items	Experimental groups^1^			SEM^4^	*P*-value^6^
	SP0	SP0.5	SP1		
Digestion coefficients, %					
Dry matter	63.77	64.20	63.97	1.44	0.783
Organic matter	65.42	65.83	65.62	0.85	0.855
Crud protein	66.08^b^	66.39^ab^	66.73 ^a^	0.28	0.033
Crude fiber	51.26	51.84	50.27	1.07	0.794
Either extract	79.67^b^	81.41^ab^	82.44 ^a^	1.06	0.038
Nitrogen-free extract	67.98	68.31	68.48	0.89	0.778
Cell wall constituents, %					
Neutral detergent fibre	55.64	56.15	55.96	0.77	0.877
Acid detergent fibre	48.64	48.84	48.88	1.06	0.894
Acid detergent lignin	38.56	39.16	39.07	1.13	0.833
Nutritive values, %					
Total digestible nutrients	64.07	64.52	64.36	0.77	0.731
Digestible crude protein	11.11	11.16	11.05	0.44	0.805
Nitrogen utilization, g/head/day					
N-Intake^5^	3.52	3.56	3.55	0.11	0.795
Urinary nitrogen	1.12	1.05	1.01	0.09	0.844
Fecal nitrogen	1.19	1.20	1.22	0.08	0.831
Absorbed nitrogen, AN	2.33	2.37	2.33	0.09	0.894
Retained nitrogen, RN	1.21^b^	1.31 ^a^	1.32 ^a^	0.05	0.016
NR/NI^2^, %	34.38^b^	36.80 ^a^	37.18 ^a^	0.36	0.031
NR/NA^3^, %	51.93^b^	55.27 ^a^	56.65 ^a^	1.22	0.017

^1^ Control: SP0; SP0.5: 0.5% Silkworm meal: SP1; 1.0% Silkworm meal

^2^ NR/NI: Ratio of retained nitrogen to nitrogen intake

^3^ NR/NA: Ratio of retained nitrogen to absorbed nitrogen

^4^ SEM: standard error of mean

^5^N-Intake = Nitrogen intake per head per day

^6^ In the rows, (*P* < 0.05) different letters (a, b, and ab) indicate statistically significant differences between means

In groups SP0.5 and SP1, nitrogen excretion in both faeces and urine, alongside digestion and retention, increased proportionately with nitrogen intake. Higher inclusion levels of SP in rabbit diets improved the connection between nitrogen intake, digestion, and retention, which was linked to body weight increase. These results are in line with those of Kowalska et al. (2020), who discovered that including 4% dry silkworm pupae and mealworm larvae meals in rabbit diets increased body weight growth relative to the control group. This was likely because the rabbits were more efficient at retaining nitrogen. It is important to note, nevertheless, that Gugołek et al. (2019) reported different outcomes.

### Carcass traits

The effects of adding SP to growing rabbits’ diets on the carcass characteristics and meat composition are displayed in Table 4. No noteworthy differences were observed in the carcass weight, dressing percentage, or slaughter body weight across all groups. Moreover, the proportions of edible organs such as the liver, kidneys, heart, lungs, and abdominal fat remained unaffected (*P* < 0.05) among the different treatments. Furthermore, the meat chemical composition in terms of protein, moisture, and ash percentages was quite similar among the groups. However, the fat percentage was considerably greater in the SP = fed rabbits (SP0.5 and SP1) than in the control group (SP0). The incorporation of SP in rabbit diets did not affect carcass characteristics and meat quality, except for meat fat %. Kowalska et al. (2020) found that carcass weight, carcass fat, and overall meat quality remained unaffected when rabbits were fed silkworm pupae. However, SP was associated with an increase in intramuscular fat and positively influenced consumer acceptability (Zubiri-Gaitán et al. 2022). Additionally, Priyadharshini et al. (2017) reported greater fat deposition and improved fur growth in rabbits that received diets supplemented with SP.

**Table 4:** Effect of silkworm (*Bombyx mori* L.) pupae meal supplementation on carcass characteristics and chemical composition of meat of growing rabbits

Items	Experimental groups^1^			SEM^2^	*P*-value^3^
	SP0	SP0.5	SP1		
Carcass characteristics					
Slaughter body weight, g	2108.3	2170.0	2170.0	122.75	0.866
Carcass weight, g	1346.3	1396.6	1399.1	96.85	0.799
Dressing, %	63.86	64.36	64.46	0.84	0.799
Edible giblets, %					
Liver	2.79	2.84	2.83	0.23	0.733
Kidney	0.77	0.76	0.76	0.11	0.875
Heart	0.39	0.39	0.42	0.09	0.855
Lung	0.48	0.47	0.48	0.06	0.795
Abdominal fat	2.76	2.99	2.89	0.36	0.744
Chemical composition of meat, %					
Moisture	72.53	72.32	72.65	1.05	0.769
Protein	22.12	22.53	22.67	1.12	0.861
Fat	4.33^b^	4.82^a^	4.94^a^	0.11	0.022
Ash	2.93	2.98	2.97	0.21	0.744

^1^ Control: SP0; SP0.5: 0.5% Silkworm meal: SP1; 1.0% Silkworm meal

^2^SEM: standard error of mean

^3^In the rows, (*P* < 0.05) different letters (a and b) indicate statistically significant differences between means

### Cecum fermentation and blood metabolites

Table 5 details how adding SP to the diets of growing rabbits affects cecum fermentation. No statistically significant changes in cecum pH were found in ammonia nitrogen (NH_3_-N), acetate, butyrate, or the acetate-to-propionate ratio across the groups. The cecal pH remained consistent across groups, with values between 6.27 and 6.30, and NH_3_-N levels also showed no notable changes, remaining close to one another. Nevertheless, the rabbits fed SP had a significantly higher level of total volatile fatty acid (TVFA) as compared with the placebo group. The groups fed SP (SP0.5 and SP1; Table 6) had significantly increased amounts of total protein, albumin, and globulin in comparison to the control group. The stability of glucose, triglycerides, cholesterol, and AST and ALT (liver enzyme) levels among all groups. Furthermore, the kidney function parameters (urea and creatinine levels) were unchanged (*P* < 0.05) across all groups. Our findings differ from those of Rashmi et al. (2022), who found that feeding DSWP did not substantially influence animal blood biochemical indicators.

**Table 5:** Effect of silkworm (*Bombyx mori* L.) pupae meal supplementation on cecal fermentation of growing rabbits

Items		Experimental groups					SEM^4^			*P*-value^5^
		SP0		SP0.5		SP1				
Caecum pH		6.27		6.30		6.29		0.11	0.688	
NH_3_-N^2^, mg/100 dL		14.12		13.82		13.96		1.31	0.794	
TVFA^3^, mmol/l		62.76^b^		63.47^a^		63.21^a^		0.35	0.022	
Acetate		65.19		65.33		65.16		0.85	0.785	
Propionate		21.38		22.52		22.49		0.53	0.772	
Butyrate		11.38		11.39		11.21		0.56	0.840	
Acetate/propionate ratio		3.05		2.90		2.89		0.27	0.737	

^1^ Control: SP0; SP0.5: 0.5% Silkworm meal: SP1; 1.0% Silkworm meal

^2^NH3-N: ammonium-Nitrogen

^3^ TVFA: total volatile fatty acid

^4^ SEM: standard error of mean

^5^ In the rows, (*P* < 0.05) different letters (a and b) indicate statistically significant differences between means

**Table 6:** Effect of silkworm (*Bombyx mori* L.) pupae meal supplementation on serum biochemical parameters of growing rabbits

Items		Experimental groups^1^						SEM^3^	*P*-value^4^	
		SP0		SP0.5		SP1				
Total protein, g/dl		6.69^b^		7.06^a^		7.16^a^		0.11		0.007
Albumin, g/dl		3.85^b^		4.13^a^		4.21^a^		0.06		0.011
Globulin, g/dl		2.84^b^		2.93^a^		2.94^a^		0.02		0.005
A/G ratio^6^		1.35^b^		1.41^a^		1.43^a^		0.01		0.001
Glucose, mg/dl		75.23		74.48		76.14		3.75		0.747
Triglycerides, mg/dl		87.72		91.10		91.31		5.55		0.873
Total Cholesterol, mg/dl		116.33		119.42		120.20		4.77		0.745
HDL^2^, mg/dl		55.82		55.99		56.32		1.64		0.794
LDL^2^, mg/dl		42.96		45.21		45.61		2.84		0.683
Urea N, mg/dl		39.85		40.26		40.87		1.07		0.842
Creatinine, mg/dl		0.82		0.82		0.83		0.33		0.868
AST^2^, U /dl		32.39		32.24		32.37		1.07		0.842
ALT^2^, U /dl		20.89		22.02		21.57		2.06		0.789

^1^ Control: SP0; SP0.5: 0.5% Silkworm meal: SP1; 1.0% Silkworm meal

^2^ A/G ratio: Albumin/ Globulin; HDL: high-density lipoprotein; LDL: low-density lipoprotein; AST: aspartate transaminase; ALT: alkaline phosphatase

^3^ SEM: standard error of mean

^4^ In the rows, (*P* < 0.05) different letters (a and b) indicate statistically significant differences between means

**Fig. 2:**
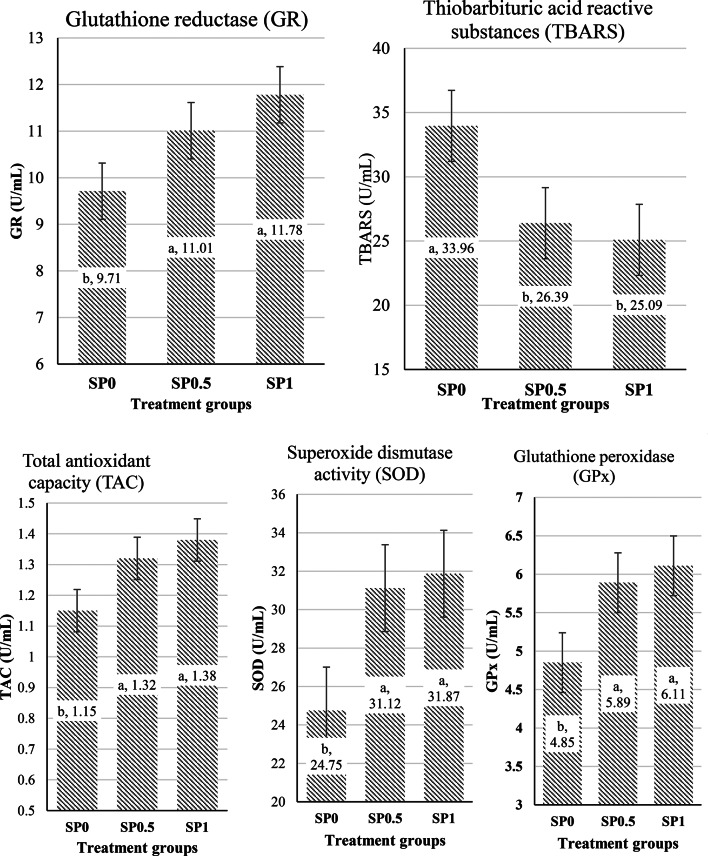
Effect of supplementing rabbits’ diet with silkworm pupae meal on blood antioxidant status. Control: SP0; 0.5% Silkworm meal: SP0.5; 1.0% Silkworm meal: SP1. Different letters (**a**, **b**) indicate statistically significant differences between means

Figure 2 represents the parameters of antioxidant capacity (TAC) and oxidative stress. TAC was markedly higher in the SP0.5 and SP1 groups. Furthermore, superoxide dismutase activity was markedly elevated in the SP0.5 and SP1 groups *versus* the control group. Similarly, the activities of glutathione peroxidase (GPx) and glutathione reductase (GR) were markedly increased in the SP groups. In contrast, the SP0.5 and SP1 groups exhibited reduced levels of thiobarbituric acid reactive compounds in comparison to the SP0 group. The use of SP led to increased blood antioxidant parameters, which could point to improvements in antioxidant ability and dealing with oxidative stress. The results correspond with the data given by Sadat et al. (2022), which show that silkworm pupae contain a varied variety of bioactive components, including antioxidants, apoptotic and antigenotoxic antitumor peptides, as well as polyphenolic compounds. Additionally, the observed reduction in thiobarbituric acid reactive substances levels suggest a decrease in cellular oxidative damage, highlighting the protective effect of SP supplementation.

### Economical assessment

The cost-benefit analysis of the addition of SP to rabbit diets is illustrated in Table 7. The primary objective is to determine whether SP supplementation is a worthwhile investment by examining the costs of nutrition, growth rates, and profits of various treatment groups. The data indicate that the cost per kilogram of feed increased as the quantity of SP was increased. Nevertheless, this was offset by the rabbits’ expanded growth. This is a significant discovery, as it demonstrates that a slight increase in feed cost is advantageous, as it results in increased production. The SP diets facilitated the weight gain of rabbits, thereby reducing the cost of weight gain per unit. This is a critical indicator of the economic efficiency of a feed composition. This indicates that the groups supplemented with SP had a higher feed conversion ratio, which implies that they required less feed to acquire a unit of weight. The statistics also indicate that the profitability of the business is positively correlated with the quantity of SP in the diet. The addition of SP increased both total and net revenue. For instance, SP0.5 generated 165.40 L.E. in total revenue and 52.47 L.E. in net revenue per capita. This represents a substantial increase from the control group, which generated 149.90 L.E. in total revenue and 40.69 L.E. in net revenue per capita. The economic advantages are further substantiated by the reported 25% increase in cost efficacy for the SP diets in comparison to the control diet. The rise in profits may be attributed to improved weight gain and potentially other factors that were not explicitly addressed, such as improved animal health or reduced mortality rates. The economic analysis suggests that the incorporation of SP into rabbit diets is a prudent economic strategy. The beneficial impact on development parameters, particularly the reduced cost of weight gain per unit, results in a substantial increase in both total and net revenue, even if the cost of feed has increased slightly. The data serve as a compelling argument in favor of the use of SP as a value-added feed supplement for rabbit farming, as it demonstrates that it is economically more advantageous than conventional diets. This demonstrates that the implementation of novel feed constituents may enhance the sustainability and profitability of rabbit husbandry.

**Table 7:** Economic efficiency of silkworm (*Bombyx mori* L.) pupae meal supplementation on rabbit diets

Item	Experimental diets^1^			SEM^3^	*P*-value
	SP0	SP0.5	SP1		
Price/kg diet^2^, L.E	17.85	18.00	18.14		
Total feed intake/rabbit, g	6123	6274	6389	153.75	0.743
Total feed cost/rabbit, L.E^A^	109.30	112.93	115.90		
Total weight gain/ rabbit, g	1363.54	1503.63	1519.8	207.74	0.683
Feed cost/ kg gain, L.E	80.16	75.11	76.26		
Total revenue/rabbit^2^, L.E ^B^	149.90	165.40	167.18		
Net revenue/ rabbit^2^, L.E ^(A-B)^	40.69	52.47	51.28		
Economic efficiency	0.37	0.46	0.44		
Relative economic efficiency, %	100	125	119		

^1^ Control: SP0; SP0.5: 0.5% Silkworm meal: SP1; 1.0% Silkworm meal

^2^ Based on the prices of the Egyptian market during the experimental period (2025). Net revenue (L. E) = (Total revenue/rabbit (L.E))- (Total feed cost/rabbit (L.E.)). Economic efficiency = (Net revenue /rabbit (L.E))/ (Total feed cost/ rabbit (L. E)). Feed cost /kg gain = Total feed cost (L.E)/ Total weight gain/rabbit (kg), the prices of live body weight of rabbits = 110 LE/kg

^3^ SEM: standard error of mean

## Conclusions

Incorporating small quantities (0.5% and 1%) of silkworm pupae meal into the diets of growing rabbits significantly enhanced their growth performance. This is due to silkworm pupae meal’s high protein and fat content, which improves nutrient digestion and nitrogen retention in the body. A substantial rise occurred in the fat percentage of the meat. The addition of SP did not influence cecal fermentation or any of the primary blood parameters. Crucially, it enhanced the rabbit’s antioxidant capacity and reduced oxidative stress, hence improving the rabbit’s health. From a business perspective, the inclusion of silkworm pupae meal was advantageous since it increased both total and net revenue per rabbit, indicating a favourable cost-benefit ratio. The results indicate that silkworm pupae meal is a durable and beneficial feed addition that enhances rabbit health, productivity, and profitability.

## Data Availability

Data will be made available on request.
